# Clinical importance of serum and pleural fluid prominin-1 and hypoxia-inducible factor-1α concentration in the evaluation of lymph node involvement in patients with malignant pleural effusion

**DOI:** 10.11613/BM.2023.030701

**Published:** 2023-10-15

**Authors:** Zeliha Cansel Ozmen, Mustafa Kupeli

**Affiliations:** 1 Department of medical biochemistry, Faculty of medicine, Tokat Gaziosmanpaşa University, Tokat, Turkey; 2 Department of thoracic surgery, Faculty of medicine, Yozgat Bozok University, Yozgat, Turkey

**Keywords:** hypoxia inducible factor-1α, malignant pleural effusion, prominin-1

## Abstract

**Introduction:**

Malignant pleural effusion (MPE) and lymph node metastasis (LNM) presence are poor prognostic factors that have importance for cancer patients. The study objective was to determine whether hypoxia-inducible factor-1α (HIF-1α) and prominin-1 (CD133) in pleural fluid (P) and serum (S) could be used as biomarkers for diagnosis of lymph node involvement in patients with MPE.

**Materials and methods:**

Fifty-six patients with MPE and 30 healthy control subjects were included. Computerized tomography (CT) and positron emission tomography (PET) were used to diagnose pleural effusion. Patients with malignant cells in pleural fluid cytological examination were included in the MPE group. Thirty-five patients with lymph node metastases on CT were included in the LNM-positive MPE group. Serum and pleural fluid HIF-1α and CD-133 concentrations were measured manually *via* enzyme-linked immunosorbent assay (ELISA).

**Results:**

Serum concentrations of HIF-1α and CD133 were higher in MPE patients. It was found that CD133/HIF-1α (S) ratio was higher in the malignant patient group with positive lymph node involvement than in the negative group, while concentrations of HIF-1α (P) were lower. Pleural fluid HIF-1α and CD133/HIF-1α (S) ratio had sufficient performance in diagnosing lymphatic metastases in patients with MPE (AUC = 0.90 and 0.83, respectively).

**Conclusions:**

In conclusion, serum HIF-1α and CD133 concentrations were higher in patients with MPE, consistent with our hypothesis. Concentrations of HIF-1α (P) and CD133/HIF-1α (S) ratio can be used as biomarkers in diagnosing lymph node involvement in MPE patients, according to this experiment.

## Introduction

Malignant pleural effusion (MPE) is defined as accumulation of significant exudates with the presence of malignant cells or tumour tissue in the pleural cavity (*1*). It is common in patients with metastatic disease and can occur in 15% of patients with cancer (*2*). Malignant pleural effusion is most commonly found in lung cancer (LC) followed by breast cancer (BC), lymphoma, gynaecological cancers, and malignant mesothelioma (*3*). Tumour cells infiltrate the pleural space through the blood, either directly or through lymphatic spread. Tumour cells in the pleural cavity can block lymphatic’s that provide fluid drainage in the parietal pleura (*4*). The inflammatory response, that develops as a result of tumour invasion to the pleura, creates effusion by increasing the pleural membrane and vascular permeability (*5*).

Prominin-1 (CD133), the first member of the prominin family, was first identified in 1997 by two independent study groups examining mouse neuroepithelial and human haematopoietic stem cells, and it was stated that prominin-1 was a common cell surface marker of both stem cells and cancer stem cells (*6*, *7*). Studies indicated that CD133 was affected by oxygen concentration (*8*, *9*). It has been shown that CD133 expression increases rapidly in hypoxic conditions (*10*). Due to the abnormal structure of tumour vessels and blood flow, the vessels become leaky, permitting the fluid accumulation in the intercellular space, thus increasing the level of hypoxia (*11*).

Transcription factors produced by cells in hypoxic conditions are hypoxia-induced factor (HIF) proteins (*12*). There are three types of HIF proteins: HIF-1, HIF-2 and HIF-3. The regulatory factor that plays a key role among these transcription factors is the HIF-1α protein (*13*). Overexpression of HIF-1α has been found to develop in response to stimuli such as hypoxia and genetic changes. While excessive HIF-1α expression is observed in 90% of lung, colon, and prostate cancers, no expression was observed in normal healthy tissues of these organs (*14*). Overexpression of HIF-1α is an important factor for tumours metabolic adaptation against hypervascularization and hypoxia. Metabolic adaptation helps tumour to gain abilities such as growth, invasion, and metastasis (*13*). In cancer, the cycle initiated by CD133 and hypoxia causes an increase in HIF-1α formation (*11*).

The presence of MPE and lymph node metastasis (LNM) are poor prognostic factors that have importance for lung cancer patients (*15*). Expression of HIF-1α and CD133 are associated with tumour progression and LNM in adenocarcinoma cells and tumour tissues (*16*, *17*). Malignant pleural effusion and lymph node involvement are among the basic criteria in TNM (primary tumour, regional lymph node, metastasis) staging system recommended by International association for the study of lung cancer (IASLC) for lung cancer and in staging system recommended by Veterans administration lung cancer study group (VALSG) for small cell lung cancer (SCLC) (*18*, *19*). This situation reveals clinical importance of the coexistence of MPE and lymph node involvement in patients with lung cancer. There has been no study examining the clinical importance of HIF-1α and CD133 concentrations in serum and pleural fluid in diagnosing lymph node involvement in patients with MPE. The objective of the present cross-sectional study was to determine whether HIF-1α and CD133 in pleural fluid and serum can be used as biomarkers in the diagnosis of lymph node involvement in patients with MPE. We hypothesized that HIF-1α and CD133 concentrations were higher in patients with MPE.

## Materials and methods

### Subjects

The study group consisted of 56 patients with MPE (31 men and 25 women, with a median age of 69 years, ranging from 36 to 96 years), who were recruited from the Hospital of Tokat Gaziosmanpasa University in 2019-2020. Thirty healthy subjects (16 men and 14 women, with a median age of 67 years, ranging from 38 to 81 years) were comparable for age, gender, without chronic or acute disease, as well as upper and lower respiratory tract infections in the previous month and were recruited as the controls from the health examination centre of the same hospital. None of the control subjects was taking any medication.

In forming the patient group, the MPE group was considered as patients with pleural effusion detected by computerized tomography (CT) and positron emission tomography (PET), with malignant cells detected by pleural fluid/bronchial lavage cytology and/or pleural tissue/bronchial tissue biopsy, or in a tissue other than pleural fluid. Patients with histologically determined malignancy or unexplained tissue with pleural effusion were included. Among this patient group, those with lymph nodes on CT were considered as the MPE group with metastatic lymph nodes. Lymph node metastasis was positive in 35 (0.63) of these patients, while negative in 21 (0.37) patients. Clinical data such as dyspnoea, chest pain, fever, and cough were collected for the patient group. Body mass index (BMI) is a simple measure of body fatness, calculated by body weight in kilograms (kg) divided by the square of height in meters (m^2^) (*20*). Body weight and height were measured in a standardized fashion between 6 and 8 a.m. following an overnight fast. The BMI of the patient and control group participants was calculated.

The study was approved by the ethical committee of our institute (Tokat Gaziosmanpasa University Clinical Research Ethical Committee; protocol number: 20-KAEK-172) and was planned and conducted according to the provisions of the Helsinki Declaration. Informed consent was obtained from each participant in the study.

### Methods

Blood was sampled by antecubital vein puncture. Pleural fluid was collected by a needle inserted between the seventh and ninth rib spaces, between the posterior axillary line and midline (thoracentesis). Both blood and pleural fluid were collected in 7 mL biochemical tubes without anticoagulants (BD vacutainer systems, Plymouth, UK). Venous blood samples were taken in the morning after 12 h overnight fasting. Pleural fluid was collected at most 2 hours after the venous blood sample was taken from the patients. After thoracentesis, the collected pleural fluid was immediately transferred into appropriate containers. Pleural fluid samples were transported to the laboratory promptly after thoracentesis at room temperature. Blood and pleural specimens were centrifuged at a relative centrifugal force (RCF) of 1917xg for 10 minutes and the supernatant was frozen and stored at - 80°C until HIF-1α and CD133 measurement were carried out, according to the manufacturer´s recommendations about tests stability.

Concentration of serum and pleural fluid CD133 and HIF-1α was measured manually *via* sandwich enzyme-linked immunosorbent assay (ELISA) (Bioassay Technology Laboratory Human Cluster of Differentiation Human Prominin-1 ELISA Kit; Shanghai YL Biotech Co., Ltd., LotNo:2001902004, Shanghai, China, and Bioassay Technology Laboratory Human Cluster of Differentiation Human-1α ELISA Kit; Shanghai YL Biotech Co., Ltd., LotNo:202205002, Shanghai, China) according to manufacturer’s instructions. The optical density was read using EPOCH 2 (Bio Tek Instruments, Winooski, USA) ELISA plate reader at 450 nm, and the results are presented as ng/mL. The concentrations were calculated using the calibrated standard curve, which was constructed using the standards provided in the kit. The minimum detectable concentration (sensitivity) for HIF-1α was < 0.01 ng/mL and detection range was 0.05 - 15.00 ng/mL. The intra-assay coefficient of variation (CV%) and inter-assay CV% values were less than 3.0% and 4.9%, respectively, as quoted by the manufacturer. The minimum detectable concentration (sensitivity) for CD-133 was < 0.051 ng/mL and detection range was 0.01 - 40.00 ng/mL. The intra-assay coefficient of variation (CV%) and inter-assay CV% values were less than 3.4% and 5.4%, respectively, as quoted by the manufacturer. In this study, the ELISA 96 well plate is coated with specific capture antibodies (against HIF-1α or CD-133). Standard solutions and samples are pipetted into the wells and the target protein presented in a sample was bound to the wells by the immobilized antibody. The wells are washed and a biotinylated detection antibody specific for the target protein is added. After washing out the unbound biotinylated antibody, horseradish peroxidase (HRP)-conjugated streptavidin is pipetted into the wells. The wells are washed again, a 3,3’,5,5’-tetramethylbenzidine (TMB) substrate solution is added to the wells, and the colour develops in proportion to the amount of bound target protein. The stop solution changes colour from blue to yellow and the absorbance in each well is measured by a microplate reader at 450 nm. The concentration of the target protein in the samples is calculated from the standard curve generated from the known concentrations and the absorbance obtained from the standard solutions used. The analyses were performed within two years of sample collection. Concentrations of HIF-1α and CD-133 were analysed by ELISA methods concurrently from the same aliquot. In addition, the entire assay was performed by the same operator.

### Statistical analysis

Data distribution patterns were tested by the Kolmogorov-Smirnov test. Normally distributed data was expressed as mean ± SD, and non-normal distributed data was expressed as mean and interquartile range (IQR). Categorical and ordinal variables were presented as numbers and ratios. Differences between the two groups were analysed with the unpaired t-test and Mann-Whitney U test. Correlations between HIF-1α and CD-133 concentrations were analysed by Spearman correlation analysis. Correlation coefficient r_s_ rs = 0.70-0.89 was considered to be strong correlation, and r_s_ = 0.40-0.69 moderate correlation (*21*). Categorical variables were expressed as frequencies and percentages and compared with Chi square test. To determine the HIF-1α and CD133 clinical usefulness in the studied group and assess the performance of the biomarker in distinguishing lymph node involvement positive and lymph node involvement negative, the marker values were reviewed by a receiver operating characteristic (ROC) analysis. The area under the ROC curve (AUC) serves as an overall measure of a biomarker/diagnostic test’s accuracy. The values P < 0.05 were considered statistically significant. Software packages SPSS version 18 (SPSS Inc., Chicago, USA) and MedCalc v.15.0 (MedCalc Software, Ostend, Belgium) were used for statistical analyses.

## Results

Demographic and clinical characteristics of the patient and control group are presented in Table 1 from which it is evident that the main cause of MPE was a lung tumour (0.55).

**Table 1:** Demographic and clinical characteristics of MPE and HC group

**Characteristics**	**MPE** **N = 56**	**HC** **N = 30**
Age, years	69 (36-96)	67 (38-81)
Gender		
Female, N (ratio)	25 (0.45)	14 (0.47)
Male, N (ratio)	31 (0.55)	16 (0.53)
BMI (kg/m^2^)	26.1 ± 5.3	29.5 ± 5.0
Primary cancer Lung cancer, N (ratio) Breast cancer, N (ratio) Colon cancer, N (ratio) Renal cancer, N (ratio) Oesophageal cancer, N (ratio) Pancreatic cancer, N (ratio) Stomach cancer, N (ratio) Ovarian cancer, N (ratio)	31 (0.55) 9 (0.16) 6 (0.10) 4 (0.07) 2 (0.04) 2 (0.04) 1 (0.02) 1 (0.02)	
Clinical symptoms Dyspnoea, N (ratio) Chest pain, N (ratio) Fever, N (ratio) Cough, N (ratio)	51 (0.91) 47 (0.84) 8 (0.14) 54 (0.96)	
BMI is presented as mean and standard deviation. BMI - body mass index. MPE - malignant pleural effusion. HC - healthy control.		

The comparison of CD133, HIF-1α and CD133-HIF-1α ratio in serum (S) of the patient and control groups are presented in Table 2. Concentrations of HIF-1α (S) and CD133 (S) were significantly higher in the MPE group compared to the control group (respectively; P < 0.001 and P = 0.018).

**Table 2:** Comparison of CD133 (S) and HIF-1α (S) concentrations and CD133/HIF-1α (S) ratios between the MPE and HC group

**Parameter**	**MPE** **N = 56**	**HC** **N = 30**	**P**
CD133, ng/mL (S)	2.22 (1.77-3.10)	1.58 (1.32-2.57)	0.018
HIF-1α, ng/mL (S)	2.11 (1.86-2.82)	1.74 (1.39-1.85)	< 0.001
CD133/HIF-1α (S)	1.12 (1.06-1.19)	0.94 (0.82-1.03)	0.264
Data are presented as median and interquartile range. CD133 - prominin-1. HIF-1α - hypoxia-inducible factor-1α. MPE - malignant pleural effusion. HC - healthy control. S - serum concentration. P < 0.05 was considered statistically significant.			

The gender distribution was 19 males and 16 females in the LNM-positive group and 12 males and 9 females in the LNM-negative group. The CD133 and HIF-1α concentrations in pleural fluid (P) and serum (S), and CD133/HIF-1α ratios in the LNM-positive group and LNM-negative patient group are presented in Table 3. The HIF-1α (P) concentrations were significantly higher in LNM-negative group than in LNM-positive group (P = 0.001). The CD133/HIF-1α (S) ratio was significantly higher in LNM-positive than in LNM-negative (P = 0.007).

**Table 3:** Comparison of the groups according to LNM presence

**Parameter**	**LNM-positive** **N = 35**	**LNM-negative** **N = 21**	**P**
Age, years	67 (45-83)	66 (49-82)	0.926
CD133 (S), ng/mL	2.4 (2.19-3.02)	2.29 (2.15-2.83)	0.522
HIF-1α (S), ng/mL	1.96 (1.82-2.17)	2.06 (1.97-2.32)	0.061
CD133/HIF-1α (S)	1.19 (0.09-1.28)	1.09 (0.75-1.16)	0.007
CD133 (P), ng/mL	4.53 (4.11-4.80)	4.11 (3.92-5.51)	0.880
HIF-1α (P), ng/mL	2.72 (2.56-3.02)	3.04 (2.98-4.13)	0.001
CD133/HIF-1α (P)	1.45 (1.37-1.69)	1.36 (1.23-1.42)	0.077
Data are presented as median and interquartile range. CD133 - prominin-1. HIF-1α - hypoxia-inducible factor-1α. LNM - lymph node metastasis. MPE - malignant pleural effusion. P - pleural fluid concentration. S - serum concentration. P < 0.05 was considered statistically significant.			

The relationship between HIF-1α (P) concentrations, CD133/HIF-1α (S) ratio and (S) and (P) HIF-1α and CD133 concentrations in LNM positive patients are presented in Table 4. A moderate correlation was found between CD133/HIF-1α (S) ratio and HIF-1α (P) (r_s_ = - 0.42, P = 0.016) and HIF-1α (P) and CD133 (P) (r_s_ = 0.51, P = 0.002). At the same time, a strong correlation was found between HIF-1α (P) and CD133 (S) concentrations (rs = 0.71, P < 0.001).

**Table 4:** Correlation between HIF-1α (P) concentration and CD133/HIF-1α (S) ratio and other parameters in the LNM-positive group

**Parameter**	**HIF-1α (P)** **N = 35**		**CD133/HIF-1α (S) ratio** **N = 35**	
	r_s_	P	r_s_	P
HIF-1α (S)	0.89	< 0.001	-	-
HIF-1α (P)	-	-	- 0.42	0.016
CD133 (P)	0.51	0.002	- 0.04	0.832
CD133 (S)	0.71	< 0.001	-	-
HIF-1α - hypoxia-inducible factor-1α. CD133 - prominin-1. MPE - malignant pleural effusion. P - pleural fluid concentration. S - serum concentration. r_s_ - correlation coefficient. P < 0.05 was considered statistically significant.				

**Figure 1:**
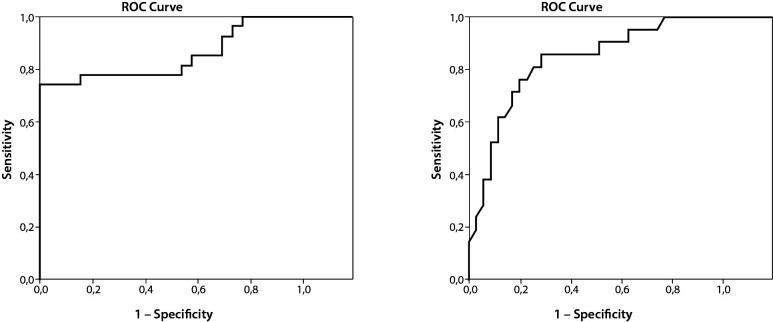
ROC analysis of HIF-1α (P) (A) and CD133/HIF-1α (S) ratio (B) in diagnosing lymphatic metastasis in patients with malignant pleural effusion. HIF-1α - hypoxia-inducible factor-1α. P - pleural fluid. CD133 - prominin-1. S - serum.

The ROC analyses of the HIF-1α (P) and CD133/HIF-1α (S) ratio are shown in Table 5 with their cut-off points and corresponding diagnostic characteristics. We selected the positivity cut-off points after performing the test, choosing the ones that maximized tumour marker performance. The best cut-off values were based on the ROC analysis performed using MedCalc software. The marker values with the highest AUCs were for HIF-1α (P) AUC = 0.83, and for CD133/HIF-1α (S) ratio AUC = 0.90. The ROC curves are presented in Figure 1.

**Table 5:** ROC analysis results for HIF-1α (P) and CD133/HIF-1α (S) ratio in diagnosing LNM in MPE patients

	**HIF-1α (P) (ng/mL)**	**P**	**CD133/HIF-1α (S) ratio**	**P**
AUC (95% CI)	0.83 (0.71-0.94)	< 0.001	0.90 (0.81-0.98)	< 0.001
Cut-off	≤ 3.04	/	≥ 1.10	/
Sensitivity (%)	81	/	86	/
Specificity (%)	74	/	86	/
PPV (%)	85	/	91	/
NPV (%)	70	/	78	/
Efficiency (%)	79	/	86	/
ROC - receiver operating characteristic. HIF-1α - hypoxia-inducible factor-1α. CD133 - prominin-1. P - pleural fluid concentration. S - serum concentration. LNM - lymph node metastasis. MPE - malignant pleural effusion. AUC - area under the receiver operating characteristic curve. CI - confidence interval. PPV - positive predictive value. NPV - negative predictive value. The cut-offs for HIF-1α and CD 133/HIF-1α (S) ratio were chosen according to Youden index. P < 0.05 was considered statistically significant.				

## Discussion

Lymph node involvement is an important borderline criterion for staging M0 patients in the International lung cancer staging project (TNM staging) (*22*, *23*). The presence of LNM is one of the criteria for the worst survival. Lymph node metastasis can be detected by CT scan in patients with MPE. A serum or pleural fluid biomarker that can detect MPE and LNM at the same time can be very useful in terms of early diagnosis and prognosis in these patients. This experiment illustrated that HIF-1α (S) and CD133 (S) concentrations are higher in MPE patients, also that HIF-1α (P) and CD133/HIF-1α (S) ratio parameters had sufficient diagnostic performance in the diagnosis of lymph node involvement.

Although CD133 functions in lung cancer is not fully elucidated, it is one of the most widely used markers of lung cancer stem cells, which are characterized by self-renewal and tumourigenicity (*17*). Numerous studies determined that CD133 cells increase in non-small cell lung carcinoma (NSCLC) and have a high tumourigenic potential. Mizugaki *et al*. demonstrated that CD133 expression was associated with poor prognosis in a study of 161 NSCLC patients, and Wu *et al*. explained in a meta-analysis conducted on 23 studies, that CD133 could be a potential prognostic marker in NSCLC (*24*, *25*). Yong *et al*. investigated the clinical significance of CD133 in a study on patients with MPE caused by lung cancer and found that CD133 could be a potential marker of advanced disease and treatment resistance (*26*). Mancini *et al*. showed in cell culture and animal experimental studies that MPE caused by lung cancer is an excellent source for cancer stem cells (CSCs), as in solid tumours (*27*). In our study, blood CD133 concentrations were evaluated as a marker in MPE patient serum, and significant difference in CD133 (S) concentrations was found between the healthy, control group and malignancy group. It has been reported that CD133 expression is associated with poor tumour differentiation and lymph node metastasis in tumour tissue, immunohistochemically determination and cell culture, and most probably poor prognosis of lung cancer (*17*, *28*). Still, it is controversial whether CD133 is associated with clinical-pathological features and prognosis of lung cancer (*17*). In this study, no significant difference was found in pleural fluid CD133 concentration in LNM-positive group. Although there was an increase in serum CD133 in MPE patients, the absence of this increase in LNM patients made us think that CD133 expression in MPE was caused by the primary tumour, not LNM. In order to explain CD133 expression from LNM in MPE patients, pleural fluid CD133+ cell content can be investigated. Conducting such a study can shed light on whether LNM is associated with CD133+ cells in MPE. Faslow *et al*. showed that CD40 (S) protein can be detected in blood taken from lung cancer patients and in pleural effusion (*29*). In their independent study confirming these results, Mu *et al*. also found that CD40 (S) concentration in advanced stages of lung cancer are significantly higher than in the early stage of the disease (*30*). Serum CD133, which is thought to be increased in NSCLC and have high tumourigenic potential, was increased in this study. However, there was no increase in both serum and pleural fluid in the case of a poor prognostic indicator such as the LNM occurrence. This result suggested that the use of CD133 alone in patients with MPE may not contribute enough to the LNM evaluation.

One of the regulatory mechanisms of CD133 expression in cancer cells is HIF-1α (*31*). Overexpression of HIF-1α in lung tumour cells is associated with higher invasiveness and metastasis, and HIF-1α is considered to be a biomarker of poor prognosis in human lung cancer (*16*). According to previous studies, higher HIF-1α concentrations have been identified in SCLC and NSCLC, and it has been revealed that these higher HIF-1α concentrations may be associated with disease stage, survival, and treatment response (*32*, *33*). It is thought that HIF-1α activation, which is a common feature of tumour cells, is more pronounced especially in aggressive tumours and may be an independent marker of poor prognosis (*34*). Zhong *et al*. investigated HIF-1α expression in 179 tumour tissues consisting of 19 different tumour types and adjacent normal tissues, and detected excessive HIF-1α expression in 13 tumour types compared to adjacent healthy normal tissue. In addition, they envisaged that this excessive HIF-1α expression may occur in early stages of carcinogenesis (*14*). Zibin *et al.* found significantly higher HIF-1α (S) concentrations in the patients with MPE and tuberculosis pleural effusion than those of the healthy control group in a study using ELISA. Also, the study found the concentration of HIF-1α (P) in the malignancy group to be significantly higher compared to the pleural fluid concentration of the group with tuberculosis effusion and thought that HIF-1α could be useful clinically in distinguishing benign and malignant effusions (*35*). In a similar study, Zang *et al* determined serum and pleural fluid HIF-1α concentrations in patients with malignant pleural effusion, benign pleural effusion, and healthy control group by ELISA. They found that HIF-1α (S) concentrations of the malignant and tuberculosis effusion group were significantly higher than the healthy control group, but did not find a significant difference between two effusion groups. In addition, the HIF-1α (P) concentration of the malignancy group was significantly higher than the group with tuberculosis effusion (*36*). In this research, we found that HIF-1α (S) concentrations were higher in MPE patients. The increase in HIF-1α (S) in these patients suggest that it may be due to lung cancer as well as cancer-related MPE. To explain this situation, it might be useful to conduct studies that include patient groups with and without MPE and to study the HIF-1α (S) concentrations. In various types of cancer, HIF-1α expression was reported to be associated with lymphatic metastasis, but the underlying mechanism has not yet been well characterized (*37*, *38*). Katsuda *et al*. found that HIF-1α expression was correlated with lymphatic invasion and vascular endothelial growth factor (VEGF-C) expression in oesophageal squamous cell carcinoma (ESCC). They think that HIF-1α plays a role in lymphatic invasion and lymph node metastasis through the induction of VEGF-C in ESCC (*39*). In this study, HIF-1α concentration was increased in serum in MPE and decreased in pleural fluid in the group with LNM. Contrary to other study results, there was no increase correlated with lymphatic metastasis. This situation made us think of two possibilities; 1) decreased pleural fluid HIF-1α expression in patients with MPE who developed lymphatic metastases 2) increased pleural fluid lymphatic drainage and/or decreased pleural fluid HIF-1α concentration due to pleural fluid volume increase. There is a need for further studies in which pleural fluid VEGF-C concentrations are measured. The fact that CD133 cells increase in cancerous tissue, induce hypoxia and cause HIF-1α expression raised the question of whether the CD133/HIF-1α ratio could also be useful as a new marker in this study (*31*). No significant change was found in CD133/HIF-1α (S) ratio in MPE patients compared to the control group, perhaps due to inadequate amount of free CD133 in the blood of cancer patients. Our aim in calculating the CD133/HIF-1α ratio was to evaluate the level of hypoxia-induced CD133 expression in MPE patients. There was a slight increase in this rate in MPE patients but this was not a significant increase. The importance of hypoxia-induced CD133 expression in MPE patients in disease diagnosis, follow-up and prognosis can be evaluated with studies that will use larger numbers of patients in this regard. CD133/HIF-1α ratio is a parameter investigated for the first time as far as we can see in our literature review.

Expression of HIF-1α were associated with tumour progression, poorly differentiated cells, histological type, tumour stage, lymph node metastasis, and poor survival (*16*, *32*). In their meta-analysis, Ren *et al.* found that HIF-1α expression was excessive in lung cancer tissues and that this overexpression was not present in healthy lung tissues. Increased CD133/HIF-1α (S) ratios in LNM positive group and moderate correlation between HIF-1α (S) and CD133 (S) concentrations and strong correlation between HIF-1α (P) and CD133 (S) suggested that a mechanism might be activated that would increase the transfer of HIF-1α from pleural fluid to plasma in case of lymph node involvement in MPE patients. One of the findings that suggest this idea was the high HIF-1α (S) concentration in this malignant patient group. At the same time, the strong correlation between HIF-1α (P) and CD133 (S) concentration in this malignant patient group reveals the importance of HIF-1α secreted at the tissue level in the increase of plasma CD133. High CD133/HIF-1α (S) ratio in the LNM-positive group showed that HIF-1α was lower in serum in the case of lymph node involvement. Our results facilitated thinking that due to the lymph node involvement in MPE, the passage of HIF-1α produced at tissue level and HIF-1α in pleural fluid into plasma may be lower.

In our literature review, we could not find an article evaluating the diagnostic importance of HIF-1α and CD133 as a biomarker in patients with MPE. Chen *et al*. reported the sensitivity and specificity for increased VEGF mRNA expression in pleural effusions as 82.6% and 84.3%, respectively (*40*). Katsuda *et al*. stated that HIF-1α may play a role in lymphatic invasion and lymph node metastasis by inducing VEGF-C in ESCC (*39*). When we evaluated HIF-1α (P) as a biomarker in the diagnosis of LNM in patients with MPE, it was thought that it could have sufficient performance with 0.83 (0.71-0.94) AUC values and 79% efficiency. The CD133/HIF-1α (S) ratio had a higher diagnostic performance than HIF-1α, with an AUC of 0.90 (0.81-0.98) and an efficiency of 86%. These results suggest that CD133/HIF-1α (S) ratio can be evaluated as a new biomarker in the diagnosis and follow-up of lymph node involvement in patients with MPE.

This study has some limitations. The sample size was relatively small, and the study was conducted at a single centre that may not be representative of MPE patients from other centres. In spite of that, the number of patients included in this study is representative of the size of the medical facility where the study was performed. In the study CD133 (P) cells could not be demonstrated. However, determination of CD133 (P) cell counts and their relationship with pleural fluid and (P) CD133 and (P) HIF-1α concentrations may help to explain the effect of CD cells increase in pleural fluid due to hypoxia on LNM. The study needs to be supported by further, multicentre studies involving a larger patient group and detecting the CD133 (P) cell content.

In conclusion, serum HIF-1α and CD133 concentrations were higher in patients with MPE, consistent with our hypothesis. However, there was no increase in serum concentration of HIF-1α and CD 133 in these patients with lymphatic involvement. Moreover, as an interesting result, lower pleural HIF-1α concentrations were found. According to this experiment, HIF-1α (P) and CD133/HIF-1α (S) ratio can be used as biomarkers in diagnosing lymph node involvement in MPE patients.
